# Everybody's Page

**Published:** 1889-11-02

**Authors:** 


### Everybody's Page.

Ripe tomatoes will remove ink and other stains from white cloth.

A new intoxicant, sugar-cane wine, has been invented, and is said to be very pleasant to the palate.

A lotion made from tobacco waste is now being manufactured in Russia, and sold for the cure of various skin diseases to which sheep are liable.

*L'Industrie Parisienne* says that in some French laundries boiled potatoes are preferred to soap for the cleansing of soiled linen.

The cinchona has been introduced into the Sandwich Islands, and is doing well. Some species give about 23lbs. of bark for every tree, and of this about 10 per cent. consist of alkaloids.

"You should sue the company for damages," said a sympathetic friend to an Irishman who had been injured in a railway accident. "Damages!" was the indignant reply, "I've enough of them already. It's repairs I'm wanting."

It was a law of the kingdom of Burmah that all rubies above a certain size should be delivered up to the Government. The result of this was that the finder of large rubies usually broke them in pieces in order to be allowed to keep them.

The German Army Medical Service has been setting its wits to work to prevent the excessive perspiration of soldiers' feet. They now recommend washing the feet with a weak solution of chromic acid once in two or three weeks. Washing the feet with soap and water once a day is also useful, but perhaps the Teuton does not like this treatment.

It was about the year 1850 that the use of iced water as a beverage became popular in America. It is now all but universal, and icemaking has become a recognised industry there. A Boston merchant, named Tudor, began to export ice in 1802, and though at first the trade did not progress rapidly, by 1847 the export from Boston alone was 51,887 tons.

The Government of Victoria are taking measures to prevent the importation and sale of opium for the purpose of smoking. A Chinese gentleman, Cheok Hong Cheong, was the first to draw the attention of the Government to the mischief wrought by the opium habit among his poor compatriots, and to ask that opium, so far as regards its sale, shall be put on the same footing as other poisons.

The Chinaman has no nerves, as nerves are understood by fidgetty Europeans and Americans. He never finds his work monotonous, or at least the monotony does not worry or irritate him. Even the Chinese schoolboy does not as a rule want to play truant and go on strike because his lessons are too long. It is this quality of stolid perseverance which enables the Chinese to compete so effectively in the labour markets of the world.

The water-lily is largely used in some parts of India as a food-stuff. The fruit of one species that grows plentifully in the lakes of Cashmere is rich in starch and has much the flavour of a chestnut. If the nuts are dried they will keep for a long time, and when ground may be made into cakes or porridge, or they may be soaked for some hours and then boiled. The seeds of the lotus also are much used in India. When green they are eaten raw; when ripe they are boiled. The root, too, is often boiled and served as a vegetable.

The smell of finely-scraped horse-radish is said to be an effectual cure for headache.

The latest device of the chiropodist is a show card with the heading "See the corn-curing hero comes."

Extracts of verbena, so-called, have no verbena in them, but are obtained from the odorous lemon grass, which grows in India.

About 100,000 tons of carbon are annually sent up London chimneys in the form of smoke, which might, by the aid of science, be retained and used as fuel.

The popular dread of night air is a very unreasonable one. The air of London is purer after ten o'clock at night than it is during the day, when a thousand chimneys are sending their smoke up to defile it.

The production of salt by the evaporation of sea-water is still largely carried on in warm countries which have an extensive seaboard. In Portugal about 250,000 tons are annually produced in that way, and about as much from the Atlantic and Mediterranean coasts of France.

Flower culture is one of the most promising industries in Florida, and it is said that as good attar of rose can be made there as in Bulgaria. The musk and the damask rose flourish there admirably, and the other flowers used in the manufacture of perfumery, such as jasmine, lilies, violets, and jonquils, grow in abundance.

Water is sometimes, though too rarely, boiled before being drunk in the family circle, but to the Sanitary Board of Houghton-le-Spring belongs the distinction of having boiled the water for a whole village before allowing it to enter the reservoir. Having traced an outbreak of enteric fever to the great Herrington well, they erected engine-boilers near it, and boiled every drop of water that was taken out.

The district from which most of the Hungarian bitter waters are now taken was, forty years ago, a large pond, on the shores of which crystals of sodium sulphide were frequently found. During the next ten years this pond was drained and the ground cultivated. It was then necessary to dig wells, and in 1863 the water from a newly-made well was found to have a salt and bitter taste, and on being analysed was proved to have a distinct medicinal value. On this there sprang up a rage for digging for mineral waters, and the Hunyadi Janos and various other bitter waters came into the market.

The first strike of schoolboys occurred, not in 1889, but in 1595, but it also occurred in rebellious Scotland. It pleased the magistrates of Edinburgh in that year to ordain that the holidays of the pupils at the High School should last only fourteen days. The free instincts of the Scottish schoolboy rebelled at this, and so, as an ancient chronicler puts it, they "made a mutinie." They went to school indeed, but they took with them weapons, ammunition, and provisions, and locked the masters out. They were prepared to stand a siege rather than sacrifice a single Saturday afternoon. The city officials went and called on the boys in a peaceable manner to open their doors. The boys did not open their doors; they fired a gun instead. It was Master William Sinclair, son of the Chancellor of Caithness, who did the deed, and, whether by chance or skill, he hit a prominent citizen, Bailie John Macmoran, with fatal effect. The window from which the shot was fired was called the "Bailie's window" as long as the old High School stood.

